# First trimester circulating miR-208b-3p and miR-26a-1-3p are relevant to the prediction of gestational hypertension

**DOI:** 10.1186/s12884-025-07349-x

**Published:** 2025-03-08

**Authors:** Andrée-Anne Clément, Cécilia LÉGARÉ, Véronique Desgagné, Kathrine Thibeault, Frédérique White, Michelle S. Scott, Pierre-Étienne Jacques, William D Fraser, Patrice Perron, Renée Guérin, Marie-France Hivert, Anne-Marie Côté, Luigi Bouchard

**Affiliations:** 1 Department of Biochemistry and Functional Genomics, Faculty of Medicine and Health Sciences (FMHS), Université de Sherbrooke, Sherbrooke, Québec Canada; 2 Plateforme de recherche, de valorisation, d’analyse et de liaison en informatique de la santé (PREVALIS), Faculty of Medicine and Health Sciences, Université de Sherbrooke, Sherbrooke, Québec Canada; 3 RNA Institute, College of Arts and Sciences, University at Albany-SUNY, Albany, NY USA; 4 School of Rehabilitation Sciences, Faculty of Medicine, Université Laval, Quebec, Canada; 5 Clinical Department of Laboratory Medicine, Pavillon des Augustines, Centre intégré universitaire de santé et de services sociaux (CIUSSS) du Saguenay–Lac-St-Jean – Hôpital Chicoutimi, Saguenay, Québec Canada; 6 Department of biology, Faculty of Sciences, Université de Sherbrooke, Sherbrooke, Québec Canada; 7 Centre de Recherche du Centre hospitalier universitaire de Sherbrooke (CR-CHUS), Sherbrooke, Québec Canada; 8 Department of Obstetrics and Gynecology, Faculty of Medecine and Health Sciences, Université de Sherbrooke, Sherbrooke, Québec Canada; 9 Department of Medicine, Faculty of Medecine and Health Sciences, Université de Sherbrooke, Sherbrooke, Québec Canada; 10 Department of Population Medicine, Harvard Pilgrim Health Care Institute, Harvard Medical School, Boston, USA; 11 Diabetes Unit, Massachusetts General Hospital, Boston, USA

**Keywords:** Pregnancy-induced hypertension, Ribo-hormones, Non-invasive biomarkers, MicroRNA (miRNA), Non-coding RNAs, Human diseases

## Abstract

**Background:**

Gestational hypertension (GH) is linked to an increased risk of cardiometabolic diseases for both mother and child, but we lack reliable biomarkers to identify high-risk women early in pregnancy. MicroRNAs (miRNAs) are small non-coding RNA that have emerged as promising biomarkers for pregnancy complications. We thus aimed to identify first trimester circulating miRNAs associated with GH and to build a miRNA-based algorithm to predict GH incidence.

**Methods:**

We quantified miRNAs using next-generation sequencing in plasma samples collected at first trimester of pregnancy in Gen3G (*N* = 413, including 28 GH cases) and 3D (*N* = 281, including 21 GH cases) prospective birth cohorts. MiRNAs associated with GH in Gen3G (identified using DESeq2, *p*-value < 0.05) and replicated in 3D were included in a stepwise logistic regression model to estimate the probability of developing GH based on the miRNAs (normalized z-score counts) and maternal characteristics that contribute most to the model.

**Results:**

We identified 28 miRNAs associated with the onset of GH later in pregnancy (*p* < 0.05) in the Gen3G cohort. Among these, three were replicated in the 3D cohort (similar fold change and *p* < 0.1) and were included in stepwise logistic regression models with GH-related risk factors. When combined with first trimester mean arterial pressure (MAP), miR-208b-3p and miR-26a-1-3p achieve an AUC of 0.803 (95%CI: 0.512–0.895) in Gen3G and 0.709 (95%CI: 0.588–0.829) in 3D. The addition of miR-208b-3p, and miR-26a-1-3p to the model significantly improves the prediction performance over that of MAP alone (*p* = 0.03). We then proposed low and high-risk thresholds, which could help identify women at very low risk of GH and those who could benefit from prevention monitoring throughout their pregnancy.

**Conclusion:**

The combination of circulating miR-208b-3p and miR-26a-1-3p with first trimester MAP offers good performance as early predictors of GH. Interestingly, these miRNAs target pathways related to the cardiovascular system and could thus be relevant to the pathophysiology of GH. These miRNAs thus provide a novel avenue to identify women at risk and could lead to even more adequate obstetrical care to reduce the risk of complications associated with GH.

**Supplementary Information:**

The online version contains supplementary material available at 10.1186/s12884-025-07349-x.

## Introduction

Early gestational physiologic adaptations are intended to meet the metabolic demands of both mothers and their growing fetuses for a successful pregnancy [1–3]. During normal pregnancy, blood pressure (BP) drops very early in the first trimester and continues to decrease until a nadir is achieved at around 22–24 weeks. Then, BP rises slowly back to pre-pregnancy levels after delivery [4]. However, abnormal BP during pregnancy is relatively common and gestational hypertension (GH) affects approximately 5% of pregnancies globally, with prevalence fluctuating according to geographical regions [5–7]. GH is defined as *de novo* hypertension corresponding to systolic blood pressure (SBP) ≥ 140 mmHg and/or diastolic blood pressure (DBP) ≥ 90 mmHg GH in the second half of pregnancy (> 20 weeks of pregnancy) [8]. It can have serious impacts during pregnancy, including a higher risk of preeclampsia (PE), intrauterine growth restriction and preterm delivery [9]. It is also well documented that higher BP in pregnancy heralds future cardiovascular and metabolic health risks for women over their life course [10–12]. Moreover, the risk of GH increases in the presence of known risk factors such as obesity, diabetes, and advanced maternal age at pregnancy onset [1].

The significant rise in BP observed in GH places excessive stress on the maternal cardiovascular system and the fetus [3, 13]. Although identification of women at higher risk of GH could help mitigate the complications of GH exposure for both the mother and her child, our capacity to assess GH risk before its onset remains limited [14]. Indeed, GH is rarely investigated alone as most studies also include PE cases, especially when studies are conducted within the first trimester of pregnancy [15]. However, while PE is usually considered more serious than GH, the latter itself also has consequences on both mother and child exposed to the condition. Evidence suggests that GH and PE share some but not all risk factors and are marked by distinct inflammatory profile at first trimester [16–18]. These differences could reflect many etiologies, underlying mechanisms, and physiological markers of these conditions. Moreover, prophylactic aspirin treatment to women at risk reduces the risk of pre-term PE mostly, having little to no effect on women at risk of GH [19–21]. Therefore, women identified at risk of GH early in pregnancy would benefit from a timely prediction that would allow to optimize the management of their pregnancy. Few early pregnancy predictors such as mean arterial pressure (MAP) and maternal characteristics considered alone or in combined algorithm are available to inform prediction models of GH [15]. However, not all women at risk of GH present classic risk factors for this condition. Early prediction using novel biomarkers could thus improve risk assessment and allow targeted interventions to prevent unnecessary adverse short and long-term outcomes related to GH.

MicroRNAs (miRNAs) are small non-coding RNA sequences of 19–24 nucleotides in length. They modulate gene expression post-transcriptionally by interfering with their messenger RNA (mRNA) targets [22]. MiRNAs are found in all biological fluids and are secreted in blood from various organs, including the placenta [23]. In blood, it has been proposed that miRNAs could have hormone-like functions impacting many physiological processes [22, 24]. When secreted by the placenta into the maternal circulation, they could play a role in feto-maternal communication mediating the many physiological adaptations to pregnancy, including maternal blood pressure regulation [25, 26]. Given their characteristics, miRNAs are both potential key factors in disease development and ideal biomarkers for predicting numerous conditions, including pregnancy complications such as gestational diabetes mellitus (GDM) and hypertensive disorders of pregnancy (HDP) [27–30]. Our objectives were to identify first trimester circulating miRNAs associated with GH weeks before the onset of GH and to assess their clinical relevance through their integration into multiparameter predictive models. We hypothesized that pregnant women with GH have a distinctive circulating miRNA profile in the first trimester of pregnancy. We also hypothesized that some of these miRNAs can be used to predict risk of GH incidence and tested the performance of our predictors in an independent pregnancy cohort.

## Materials and methods

### Participants’ selection – discovery cohort

Participants of the discovery cohort were selected among the Genetics of Glucose regulation in Gestation and Growth (Gen3G) prospective birth cohort [31]. Women were included in the Gen3G cohort if they were ≥ 18 years old with a singleton pregnancy. Women were excluded if they took medication that could influenced glycemia, had pre-pregnancy diabetes or diabetes diagnosed at the first visit (between 4 and 16 weeks of pregnancy). For this study, women were included if plasma samples at first visit, complete oral glucose tolerance test (OGTT) data at the second visit (between 24 and 28 weeks of pregnancy), both mother and child follow-up until 5-years post-partum and genetic and epigenetic data were available. We excluded women with chronic hypertension or other pre-existing conditions such as polycystic ovarian syndrome and hypercholesterolemia, but women with gestational diabetes mellitus (GDM; *n* = 58) diagnosed at the second visit remained in our study sample. In total, 444 women of European descent, including 29 GH cases fulfilled our selection criteria and were included (Supplementary Fig. 1). Each woman provided informed consent and this work was approved by the ethics review board of the CIUSSS de l’Estrie-CHUS.

### Participants’ selection – replication cohort

Participants for our replication were selected from the 3D (Design, Develop, Discovery) prospective pregnancy and birth cohort [32]. Women were included if they were of European descent without a diagnosis of pre-gestational diabetes (either Type 1 or 2) or chronic hypertension without further PE diagnosis at the first trimester of pregnancy to ensure comparison with the Gen3G discovery cohort. Participants were selected based on availability of plasma samples at first visit (between 6 and 15 weeks of pregnancy) and OGTT data (at fasting and 2 h post-OGTT) at second visit (between 24 and 28 weeks of pregnancy). A total of 317 women, including 21 GH cases fulfilled our selection criteria. All women provided written informed consent in accordance with the Declaration of Helsinki and the Research ethics committee of the Sainte-Justine University Hospital Centre approved the 3D cohort and the CIUSSS de l’Estrie-CHUS ethics board approved this study.

### GH and GDM classification in Gen3G and 3D cohorts

GH was diagnosed according to the Society of Obstetricians and Gynaecologists of Canada (SOGC) guidelines, which defined new-onset hypertension as at least two measures of systolic blood pressure (SBP) ≥ 140mmHg and/or diastolic blood pressure (DBP) ≥ 90mmHg after 20 weeks of pregnancy [33]. Women were considered normotensive if they did not meet these clinical thresholds. GH cases were reviewed and discussed with an obstetric nephrologist (AM Côté). Women who developed PE (corresponding to GH with proteinuria) were not included in this current analysis (Gen3G, *N* = 22; 3D, *N* = 27). GDM diagnosis was based on OGTT results according to the International Association of Diabetes and Pregnancy Study Groups (IADPSG) [34].

### Clinical and anthropometric measurements

Clinical and anthropometric variables were measured using standardized protocols and procedures as previously reported for both cohorts [31, 32]. At the first visit, SBP and DBP (in mmHg) were measured (thrice in Gen3G and twice in 3D) in a sitting position after 5 min of rest, as recommended. Mean SBP and DBP were computed and used in our analyses. MAP was calculated as the sum of SBP and two times DBP, divided by three [35, 36]. Clinical, anthropometric, and biochemical assessments were repeated at a second visit (between 24 and 28 weeks of pregnancy). End of pregnancy and delivery data were collected from hospital data records (including BP measurements and proteinuria results) [31, 32].

### RNA extraction and library preparation for Gen3G and 3D cohort

RNA was extracted from 500µL of plasma collected during first trimester using the mirVana Paris kit (Thermofisher Scientific, catalogue # AM1556) and according to the manufacturer standard protocol for total RNA isolation [25]. Following the modified procedure established by Burgos et al., RNA was eluted in 75 µl of nuclease-free water and concentrated by precipitation [25, 37]. Libraries were prepared using the entire volume of precipitated RNA using the TruSeq Small RNA Sample Prep kit (Illumina, catalogue # RS-200-0012/RS-200-0048) following an adapted version of the procedure [30, 37]. Purification, size-selection (145–160 bp) and precipitation of the libraries was done as described previously [25].

### Library quality control and miRNA sequencing

Library quality control, quantification, pooling and sequencing was performed at the McGill University and Génome Québec Innovation Centre (Québec, Canada) as previously described [25, 30]. Library quality (i.e., concentration, library length and primer dimers absence) was assessed on the Agilent 2100 Bioanalyzer and Agilent High Sensitivity DNA Kit (Agilent, Mississauga, ON, Canada; catalog #5067 − 4626) or the Kapa Illumina GA with Revised Primers-SYBR Fast Universal kit (Kapa Biosystems) and a LabChip GX (PerkinElmer, catalog #CLS760672) instrument. Samples from the Gen3G cohort were pooled equimolarly (platform HiSeq 2500: total of 56 samples sequenced at 7 pM final molarity, 12 libraries with single-index per lane; platform HiSeq 4000: total of 388 samples sequenced at 10 pM final molarity, 20 libraries with single-index per lane), denatured and clustered on an Illumina single-read flow cells (catalog #GD-401-3001 and catalog #GD-410-1001). Sequencing (single-end mode) was performed with 50 cycles, and 7 cycles of indexing read for both HiSeq 2500 and 4000. Due to the change in sequencing platform between our pilot and full project, twelves samples were extracted twice and sequenced in duplicate on both Illumina platforms to assess concordance between both platforms. Pearson correlation coefficient between both platforms was ≥ 0.94 [25]. Samples from the 3D cohort were pooled (platform NovaSeq 6000: total of 318 samples sequenced at 225 pM, 48 libraries per lane) on an Illumina NovaSeq S1 lane and sequenced for 1 × 100 cycles (single-end mode).

### Small RNA sequencing data processing

MiRNA sequencing data were processed with the extra-cellular RNA processing toolkit (exceRpt) v4.6.3 to map high-quality reads to the human genome (GRCh37) and miRBase version 21 using STAR [38–40]. In the Gen3G cohort, 8 samples were considered outliers and removed from the analysis after visualization of raw data (7 samples with < 500 000, and one with > 25 M read counts). In the 3D cohort, 12 samples with overall lower read counts (< 1 M) were considered outliers and were then also excluded from the analyses [25].

### Statistical analysis

Cohorts were compared using the χ2 test for categorical variables while the Mann-Whitney U test was applied for continuous variables. MiRNAs associated with GH were identified using the R/Bioconductor package DESeq2 using default parameters (Wald test and False discovery rate (FDR-) adjusted *p*-value) [41]. The DESeq2 implemented normalization method was used to obtain miRNAs normalized read counts which were used in the prediction modeling. Models were adjusted for batch effects (sequencing lane and run) and gestational age at first visit. miRNAs identified with a nominal *p*-value ≤ 0.05 were considered differentially abundant. The function collapseReplicates within DESeq2 was applied to combine data from samples that were sequenced in duplicate on both Illumina platforms (HiSeq 2500 and HiSeq 4000; *N* = 12). The volcano plot was built with the EnhancedVolcano package [42]. The pROC package was used to build receiver operating characteristics (ROC) curves based on the specificity and sensitivity of the model and to compute the area under the curve (AUC), an indication of the overall performance of the prediction model [43]. The statistical difference between predictive models was assessed with the likelihood ratio test while the AUCs were compared with the DeLong test. Tjur’s R^2^ (coefficient of determination) for mixed models with binary outcomes was obtained with sjPlot R package [44]. R v4.0.2 with RStudio v.1.3.10 were used to perform all statistical analyses [45, 46].

### MiRNA replication and first trimester prediction modeling of gestational hypertension

Women with complete phenotype and microtranscriptomic data were included in the prediction models (Gen3G: *n* = 384 normotensives and 28 GH; 3D: *n* = 249 and 20 GH). We considered miRNA association with GH replicated when they fulfilled the following criteria: (1) same direction of association in both cohorts; (2) achieving a nominal *p*-value < 0.1 in 3D cohort. Z-score transformation was applied to standardize miRNA normalized counts between both cohorts and allow to directly apply the equation from Gen3G to 3D. Replicated miRNAs and classic risk factors of GH were computed into stepwise logistic regression models (with direction parameter set to “both”) in the Gen3G cohort where the Akaike Information Criteria (AIC) was used to select the best variables. Each algorithm was then applied to the 3D cohort. Low and high-risk thresholds for GH risk were set by ranking the predicted probabilities obtained from the combined model with factors (clinical and miRNAs) selected by the stepwise logistic regression in the Gen3G cohort. The low-risk threshold was set to exclude all women who develop GH (sensitivity of 100%) with a 10% margin, while the high-risk threshold was established by including as many GH cases as possible while retaining an appropriate sensitivity. Both thresholds were first set in the Gen3G cohort and the cut-off values were then directly applied to 3D to assess their performance in this independent cohort.

## Results

### Demographics of the cohorts

Table 1 presents anthropometric and clinical variables of women from both cohorts. All women were considered normotensive at first trimester but those who develop GH already had higher SBP, DBP and MAP than those who remained normotensive throughout pregnancy (Gen3G: mean BP 109/68 for normotensive vs. 120/75 for GH cases; 3D: mean BP 103/62 normotensive vs. 110/67 for GH cases). In the 3D cohort only, first trimester body mass index (BMI) was higher in women who develop GH than in normotensive women. Compared to Gen3G, women from 3D were older (28.6 ± 4.2 years old in Gen3G vs. 30.9 ± 4.2 years old in 3D; *p* = 3.95e-12), more advanced in their pregnancy (9.65 ± 2.28 weeks in Gen3G vs. 11.9 ± 1.46 weeks in 3D; *p* = 2.2e-16) and their MAP was lower (82.9 ± 7.1 weeks in Gen3G vs. 76.2 ± 7.3 weeks in 3D; *p* = 2.2e-16) at first trimester visit.

**Table 1:** Characteristics of women with normotensive pregnancies and pregnancies with gestational hypertension in Gen3G and 3D cohorts

	Gen3G cohort			3D cohort		
	Normotensive *N* = 385 Mean ± SD or N (%)	GH *N* = 28 Mean ± SD or N (%)	*P*-value*	Normotensive *N* = 260 Mean ± SD or N (%)	GH *N* = 21 Mean ± SD or N (%)	*P*-value*
Age (years)	28.6 ± 4.2	27.5 ± 4.2	0.23	31.0 ± 4.2	29.5 ± 4.6	0.11
Parity (% primiparous)	197 (51%)	18 (64%)	0.25	152 (58%)	16 (76%)	0.17
Gestational age at visit 1 (weeks)	9.69 ± 2.29	9.05 ± 2.08	0.17	11.9 ± 15	12.3 ± 1.6	0.23
BMI at visit 1 (kg/m^2^)	25.7 ± 5.8	27.2 ± 5.9	0.14	24.9 ± 5.3	30.3 ± 8.6	0.002
SBP at visit 1 (mm/Hg)	109 ± 9	120 ± 9	7.07e-07	103 ± 9	110 ± 11	0.005
DBP at visit 1 (mm/Hg)	68 ± 66	75 ± 7	8.14e-06	62 ± 7	67 ± 8	0.03
MAP at visit 1	82.4 ± 6.7	90.1 ± 7.4	8.80e-07	75.7 ± 7.1	81.3 ± 8.2	0.005
GDM diagnosis (% GDM)	50 (13%)	2 (7%)	0.55	63 (24%)	5 (24%)	0.96

Data are presented as mean ± SD, unless otherwise stated. *= Comparison between normotensive and GH in both cohorts made with Mann-Whitney U test for continuous variables and χ^2^ test for categorical variables. Data were missing for: (1) Age for 1 normotensive woman in 3D (0.4%), (2) BMI at visit 1 for 4 normotensives (1.5%) and 1 GH (4.8%) in 3D, (3) SBP, DBP and MAP at visit 1 for 1 normotensive (0.3%) in Gen3G, 11 normotensives (4.2%) and 1 GH (4.8%) in 3D, (4) GDM diagnosis for 145 normotensives (55.8%) and 13 GH (61.9%) in 3D. Abbreviation: BMI: Body mass index, MAP: Mean arterial pressure, GDM: Gestational diabetes mellitus, GH: Gestational hypertension

### Selection and replication of first trimester circulating miRNAs associated with GH

**Fig. 1:**
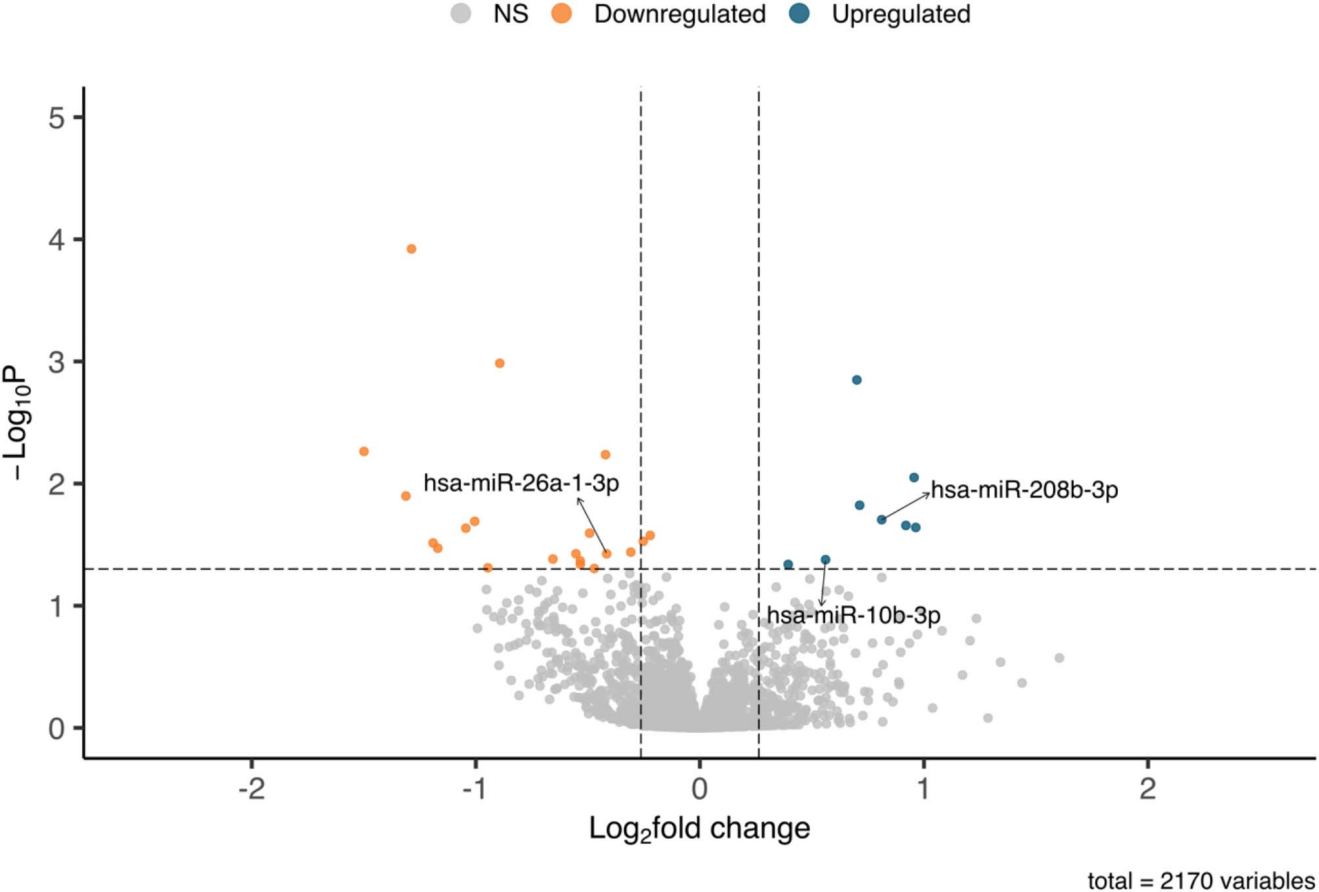
Volcano plot of the 2 170 plasma miRNAs detected by next-generation sequencing. The volcano plot shows the Log2(Fold Change) on the x-axis while the y-axis shows the significance (nominal *p*-value) as -Log_10_(*p*-value). The vertical dotted lines represent a fold change of 20% while the horizontal dotted line corresponds to a nominal *p*-value = 0.05. The 20 miRNAs less abundant in women who develop GH are represented in orange while the 8 miRNAs more abundant in women who develop GH are in blue. The three replicated miRNAs in 3D are labelled on the plot. The volcano plot was generated using EnhancedVolcano package in R (35)

In Gen3G, 2,170 unique plasmatic miRNAs were detected and quantified by next-generation sequencing [25]. The Volcano Plot (Fig. 1) shows the 28 miRNAs differentially abundant at first trimester between normotensive pregnancies and GH cases statistical significance (*p* < 0.05). The list of 28 miRNAs along with their detection rate, mean normalized counts, Log_2_(Fold Change; FC) and *p*-values are available in Supplementary Table 1. Among these, 26 had at least 20% FC between groups and are illustrated on either side of the vertical dotted lines in Fig. 1: 8 were more abundant [Log_2_(FC): 0.39–0.96] and 18 were less abundant [Log_2_(FC): -1.15 - -0.31] in women who develop GH compared to women who remained normotensive. According to our predetermined criteria, three miRNAs were considered replicated based on their same direction of association in both cohorts, i.e., a positive Log_2_(FC) for both miR-208b-3p and miR-10b-3p, and a negative Log_2_(FC) value for miR-26a-1-3p. Secondly, all three miRNAs had *p*-value < 0.05 in Gen3G and *p*-value < 0.1 in 3D (Fig. 1 – labelled, Table 2).

**Table 2:** MiRNAs associated with GH identified in the Gen3G cohort and replicated in the 3D cohort

	Gen3G cohort				3D cohort			
miRNAs	Detection rate (%) in the overall sample (*N* = 413)	DESeq2 normalized counts in the overall samples mean ± SD	Differential levels between GH and normotensive Log_2_(FC) ± SE	*p*-value	Detection rate (%) (*N* = 281)	DESeq2 normalized mean ± SD	Log_2_(FC) ± SE	*p*-value
hsa-miR-208b-3p	73.61	3.78 ± 7.17	0.81 ± 0.35	0.02	76.87	8.01 ± 16.29	0.79 ± 0.45	0.08
hsa-miR-26a-1-3p	94.92	7.15 ± 5.09	-0.42 ± 0.2	0.04	86.12	11.14 ± 12.16	-0.53 ± 0.3	0.08
hsa-miR-10b-3p	83.05	3.51 ± 3.74	0.56 ± 0.28	0.04	90.39	13.41 ± 15.24	0.88 ± 0.31	0.005

Note: Established criteria for replicated miRNAs for GH comparing to normotensive pregnancies: among miRNA differential levels achieving a *p*-value ≤ 0.05 in Gen3G, miRNA differential levels in 3D analyses should demonstrate (1) same direction of association (2) achieving a *p*-value ≤ 0.1 in the 3D cohort. Abbreviations: Log_2_(FC): Log_2_(Fold Change); SD: standard deviation; SE: standard error

### Prediction modeling analyses

#### miR-10b-3p alone performed modestly to predict GH

**Fig. 2:**
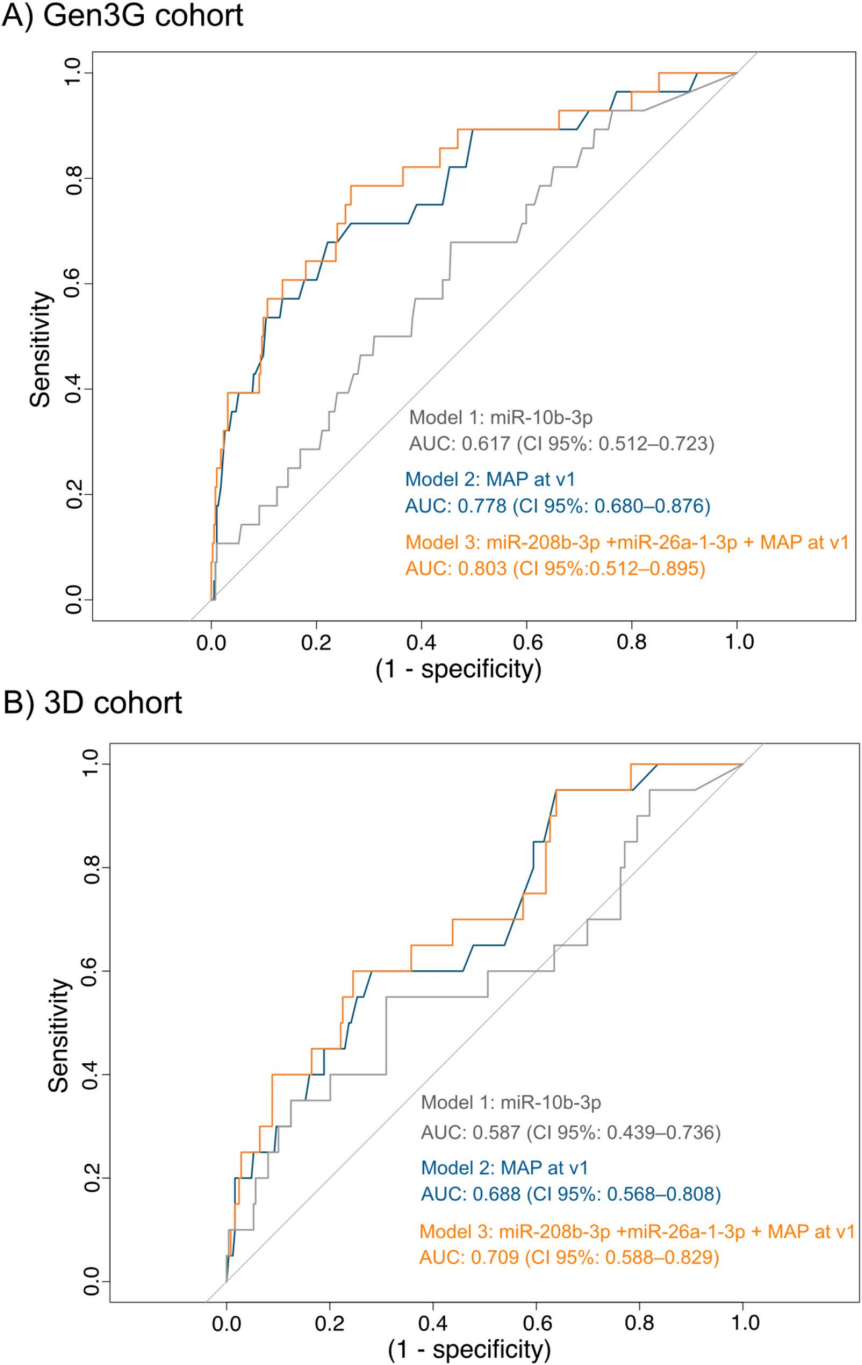
Performance of GH prediction models by Receiver Operating Characteristics (ROC) curves. (**A**) ROC curves for each model in the Gen3G cohort (*N* = 384 normotensives and 28 GH),(**B**) ROC curves for each model in the 3D cohort (*N* = 249 normotensives and 20 GH). Abbreviations: AUC: area under the curve, CI: confidence interval, MAP: mean arterial pressure at v1 (visit 1, first trimester)

A stepwise logistic regression model with the three replicated miRNAs as potential selection variables was first computed to assess their potential predictive value for GH (Model 1, Table 3). Only miR-10b-3p was selected as a predictor in the final model. Although statistically significant, this model performed modestly, achieving an AUC of 0.617 (CI 95%: 0.512–0.723) in Gen3G (Fig. 2A). In 3D, miR-10b-3p alone performed poorly, the AUC reaching only 0.587 (CI 95%: 0.439–0.736) (Fig. 2B). We assessed the predictive value of available GH classic risk factors: in this model, we included maternal age, gestational age, BMI and MAP at first visit for potential selection in the stepwise regression analyses (Model 2, Table 3). Only first trimester MAP was selected in the model and performed significantly better than the miR-10-3p-only model, with an AUC of 0.778 (CI 95%: 0.680–0.876; Fig. 2A) in Gen3G and of 0.688 (CI 95% 0.568–0.808; Fig. 2B) in 3D.

**Table 3:** Stepwise logistic regression analyses for estimated models for the prediction of GH in Gen3G

	miRNAs model (Model 1)			Risk factors model (Model 2)			Combined model (Model 3)*		
Coefficients	Estimates	Conf. Int (95%)	*p*-value^b^	Estimates	Conf. Int (95%)	*p*-value^b^	Estimates	Conf. Int (95%)	*p*-value^b^
hsa-miR-10b-3p^a^	0.32	0.00–0.60	**3.28e-02**	-	-	-	-	-	-
MAP at v1	-	-	-	0.16	0.10–0.23	**2.08e-07**	0.17	0.11–0.24	**1.26e-07**
hsa-miR-208b-3p^a^	-	-	-	-	-		0.24	-0.02–0.52	5.60e-02
hsa-miR-26a-1-3p^a^	-	-	-	-	-	-	-0.38	-0.92–0.08	1.37e-01
Observations (N)	412			412			412		
R^2^ Tjur^c^	0.011			0.109			0.144		

Variables entered in each model: (1) miRNAs model: hsa-miR-10b-3p, hsa-miR-208b-3p, hsa-miR-26a-1-3p; (2) Risk factors model: maternal age at v1; gestational age at v1; Body mass Index at v1; MAP at v1; (3) Combined model: hsa-miR-10b-3p, hsa-miR-208b-3p, hsa-miR-26a-1-3p; maternal age at v1; gestational age at v1; BMI at v1; MAP at v1. ^a^ z-score from normalized DESeq2 read counts. ^b^Variables considered as best predictors for the model are selected based on the Akaike Informative Criterion. ^c^ R^2^ Tjur (coefficient of determination, i.e., similar to pseudo R^2^) was obtained with *sjPlot* R package [41]. *Likelihood ratio test between risk factor model and combined model: *p*-value = 0.029

#### miR-208b-3p, miR-26a-1-3p and MAP improve the prediction of GH

We then combined the three miRNAs and the known clinical risk factors as potential variables for selection by the stepwise regression model to assess whether we could improve the performance of the model (based on the AUC) to predict GH incidence (Model 3, Table 3). MiR-208b-3p, miR-26a-1-3p and MAP, all measured before the 16th week of pregnancy, were considered the best predictors of GH with an AUC of 0.803 (CI 95%: 0.512–0.895) in Gen3G. This combined model was significantly better to predict GH than MAP alone (likelihood ratio test; *p*-value = 0.03) meaning that the addition of both miRNAs to the MAP improved the predictive performance of the model in the discovery cohort (Gen3G; Fig. 2A). This can be observed in Fig. 2 panel A, as the ROC curve for model 3 is slightly but noticeably shifted to the upper left when compared to the model 2 ROC curve, for specificity values between 0.5 and 0.8. However, this increase in AUC was not statistically significant (DeLong test, *p* = 0.1) The performance of the combined model to predict GH was also improved in 3D with an increased AUC of 0.709 (CI 95%: 0.588–0.829), although this gain was not statistically significant (DeLong test, *p* = 0.1) (Fig. 2B). In a similar way to Gen3G, the model 3 ROC curve in the 3D cohort (Fig. 2B) was modestly shifted to the upper left when compared to model 2.

**Fig. 3:**
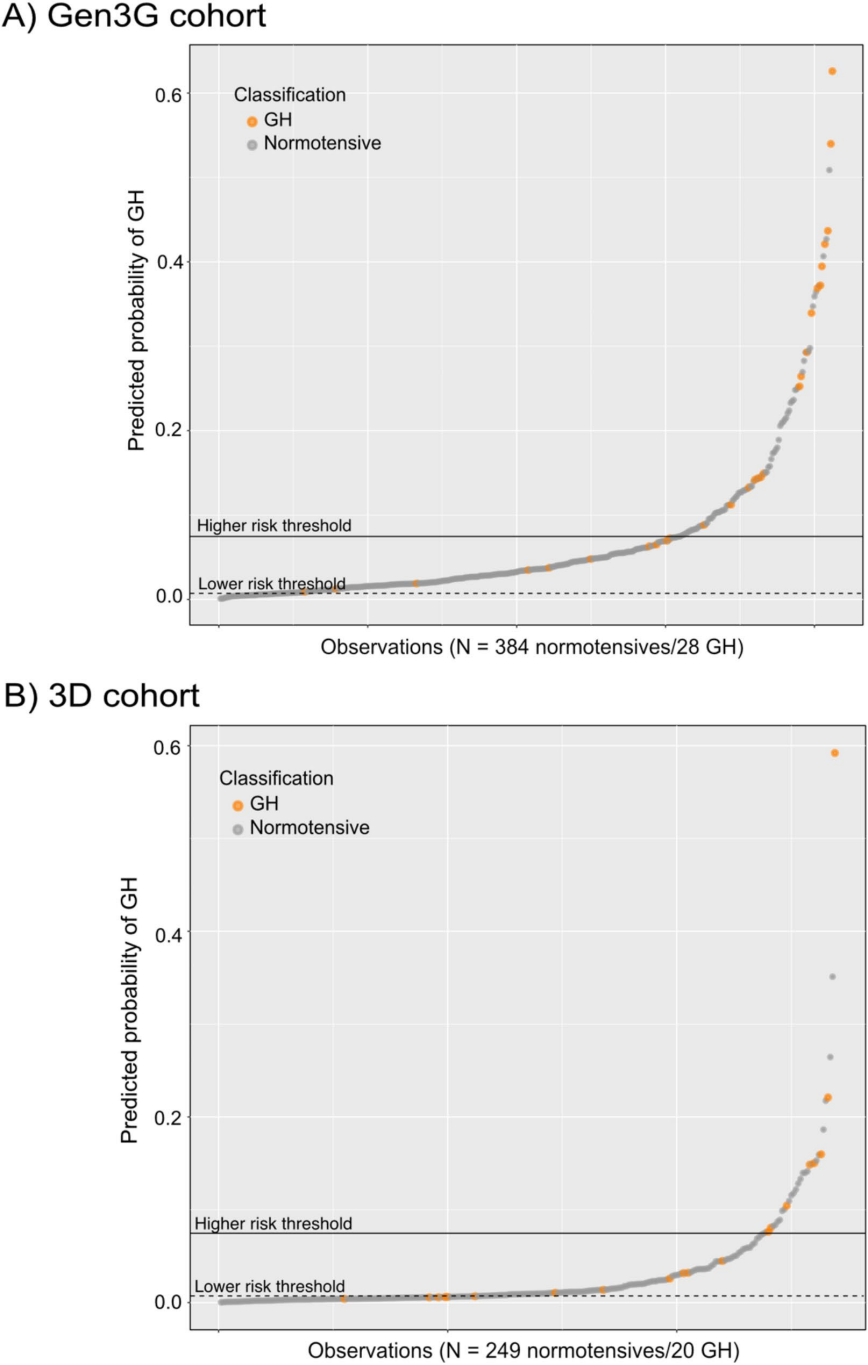
Stratification of women according to their risk of developing GH based on predicted probabilities obtained from the logistic regression model incorporating miRNAs and mean arterial pressure. Each dot represents one woman, corresponding to the number of observations on the x-axis. The predicted probabilities resulting from the logistic regression model is shown on the y-axis. The proposed high and low-risk thresholds are represented by the solid and dotted lines, respectively. **(A)** Predicted probability of GH for each woman in the Gen3G cohort; **(B)** Predicted probability of GH for each woman in the 3D cohort

To assess the potential clinical values of our prediction model, we arbitrarily selected low- and high-risk thresholds for GH in the Gen3G cohort and tested their effectiveness in both cohorts. These values were set based on the results from the logistic regression algorithm incorporating miR-208b-3p, miR-26a-1-3p and MAP. The purpose of the low-risk threshold was to exclude all women with GH (specificity of 100%), with a safety margin. The high-risk threshold was intended to identify as many GH cases as possible while maintaining an appropriate sensitivity. The selected low-risk threshold in the Gen3G cohort (cut-off value < 7.17e-3, corresponding to the 10% lowest threshold values) resulted in the exclusion of all women with GH, which can be identified below the horizontal dot line in Fig. 3A. At the high-risk threshold in the Gen3G cohort (cut-off value > 0.0747, corresponding to the upper quartile (25%) of the distribution of values), the specificity was 0.779 and the sensitivity was 0.643. Accordingly, when applying this high-risk threshold in Gen3G, we predicted 18 out of 28 GH cases (64%), corresponding to the orange dots above the solid horizontal line in Fig. 3A. The cut-off values of each threshold were then directly applied in the 3D cohort. At the low-risk threshold, the sensitivity was 0.7 and the specificity was 0.434 in 3D. Meanwhile, the high-risk threshold had a sensitivity of 0.4 and a specificity of 0.908, which translates into the identification of 8/20 GH cases in 3D (40%) (Fig. 3B).

## Discussion

In this study, we identified three circulating miRNAs quantified at first trimester of pregnancy associated with the onset of GH. Considering miRNA alone in a predictive model did not perform well. However, the best predictive performance was obtained by combining miR-208b-3p and miR-26a-1-3p with first trimester MAP. Although this is not the first attempt to identify miRNAs predictive of GH, we are the first to report, to the best of our knowledge a model combining miRNA levels with classical risk factors of GH [15, 27, 28, 47]. While none of these three miRNAs were previously reported to be associated with GH, changes in their abundance could provide additional insight into the early pathophysiology of this condition.

MiR-10b-3p was previously identified in studies on PE, but its association was opposite (downregulated) to what we found in GH [48, 49]. The difference in the direction of effect could be explained by the timing of collection (first trimester vs. delivery) or the contribution of other organs to plasma abundance and not only the placenta. It is also plausible that the targets of miR-10b-3p differ in the development of GH and PE. In PE, miR-10b-3p targeted *Lipopolysaccharide (LPS)-induced tumor necrosis factor α factor* (*LITAF*), an activator of pro-inflammatory response [49, 50]. Our study found that women with GH had higher levels of circulating miR-10b-3p then normotensive women. We could speculate that the higher miR-10b-3p abundance in GH could lead to a decrease in *LITAF* and could contribute to alleviate the inflammatory response. Further functional studies may provide more insights into the other targets and specific roles of miR-10b-3p in GH.

According to miRTarBase, miR-208b-3p targets both calcium voltage-gated channel subunit alpha1 C *(CACNA1C)* and calcium voltage-gated channel auxiliary subunit beta2 (*CACNB2)* genes encoding for subunits of voltage-dependent calcium channel proteins [51–54]. It is well documented that an imbalance in calcium homeostasis is associated with the development of hypertension, and calcium channel blockers are widely used anti-hypertensive drugs [55–57]. In this study, we report higher miR-208b-3p levels in GH cases. As miR-208b-3p appears to downregulate *CACNA1C* and *CACNB2*, it is therefore possible that it affects the number of functional calcium channels (anti-hypertensive effect) and could be seen as a compensatory mechanism attenuating the GH phenotype. We could hypothesize that this counterregulatory mechanism is ineffective to prevent GH in some women. Previous work has linked miR-208b-3p to cardiovascular injury as it was upregulated in plasma of patients with acute myocardial injury [51]. However and contrary to our study, miR-208b-3p expression was not upregulated in hypertensive patients or in atrial tissue samples collected from patients with chronic fibrillation [51, 52]. Specifically in pregnancy, we could thus speculate that miR-208b-3p changes may also relate to cardiovascular injury linked to the development of GH.

The third miRNA identified in our study is miR-26a-1-3p; a member of the miR-26 family which is involved in cardiovascular diseases through mediation of angiogenesis, endothelial cell apoptosis and left ventricular (LV) function [58, 59]. Members of the miR-26 family also display a protective role in atherosclerosis, and their expression in endothelial cells and cardiomyocytes was downregulated in models of atherosclerosis, cardiac hypertrophy and atrial fibrillation [58, 60, 61]. Compared to normotensive pregnancy, GH cases display both modified LV structure and function, including an increase in total vascular resistance and in LV mass and wall thickness [62]. MiRNA families are known to share certain targets and are therefore involved in similar biological pathways [63]. It is thus possible that the lower abundance of miR-26a-1-3p in early GH cases may be involved in the structural and functional changes observed in the LV of pregnant women who developed GH. In addition to their putative roles in cardiovascular function, both miR-208b-3p and miR-26a-1-3p directly target *tumor protein p53* (*TP53)* and *cyclin dependent kinase inhibitor 1 A* (*CDKN1A)*, also known as p21, a target of p53 [54, 64]. These two genes are primordial in cell cycle regulation and their interaction is essential for maintaining cellular integrity. Briefly, a link can be established between p53 signaling pathway and vascular and endothelial function, although its role is not yet fully understood [64, 65]. In GH, deregulation of TP53 and CDKN1A could be associated with abnormal cell cycles disrupting endothelial function, vascular remodeling and the inflammatory response, in turn targeting molecules involved in BP regulation, e.g., nitric oxide [64]. Although the association between miR-208b-3p and GH (positive) is inverse to that of miR-26a-1-3p (negative), these two miRNAs could act synergistically by targeting different players within the p53 signalling pathway which would disrupt endothelial and vascular function [66]. However, this remains a suggested hypothesis that would need to be tested in the context of functional studies to assess the role of the miRNA in GH. While these prior studies shared highlights on potential pathophysiological pathways, the three miRNAs identified in our study could contribute to GH pathophysiology through other pathways as most miRNA endocrine roles and cellular targets remain to be elucidated.

We observed that the three replicated miRNAs had abundance of a least 20% FC between normotensive and GH women. This might look like a small difference but from a clinical perspective, the increase in BP from normal values (120/80 mmHg) to a hypertensive state (140/90mmHg), corresponds to a fold change of 16% in SBP and 12.5% in DBP. While this example is biologically significant, this remains to be tested whether a 20% FC in miRNA abundance is also biologically relevant to GH. Still, this reinforces the hypothesis that modest variation in circulating miRNAs early in the first trimester of pregnancy could contribute to the pathophysiology of GH [25].

Previous studies have identified miRNAs associated with GH in clusters on chromosome 14 (C14MC) and chromosome 19 (C19MC and miR-371-3), which are known to be preferentially expressed during pregnancy [25–27, 67]. None of the three replicated miRNAs and neither of the other 25 miRNAs associated with GH in Gen3G alone were from these pregnancy-associated clusters. However, the placenta also expresses miRNAs that are not part of these clusters [26]. The miRNA identified in this study could thus still originate from the placenta or be secreted from maternal organs and tissues, in response to pregnancy or placental signals. The latter hypothesis may help to explain in part the potential role of miR-208b-3p and miR-26a-1-3p in early GH as both miRNAs were related to the cardiovascular system, which undergoes rapid change early in pregnancy in response to the action of prostacyclin, nitric oxide, and progesterone being secreted by the maternal organs and the placenta [68].

We also attempted to identify thresholds that could help to rapidly classify women at high risk of GH. These women could benefit from early counseling during their obstetrical follow-up or even antihypertensive treatments earlier in pregnancy, according to the clinician’s assessment [8]. Additionally, self-monitoring of BP could be recommended to these women early in their pregnancy [8].

### Limitation of the study

Our study has significant strengths including the identification and quantification of plasma miRNAs using next-generation sequencing in two large prospective cohorts of pregnant women totalising 694 tested samples. Our analytical strategy also included state-of-the-art replication of the results in a completely independent cohort which improved the overall validity of the results as well as our GH predictive algorithm. We acknowledge that our results did not reach statistical significance after correction for multiple testing (FDR q-value < 0.1) within each of the two independent cohorts. However, the replication-based results showed acceptable robustness, which translated into three miRNAs out of 28 with the same direction of association (Log_2_(FC)) in both cohorts and having a *p*-value below our preselected threshold that were associated with GH. Although it supports GH predictive algorithms combining miRNAs and risk factors that could be used in a clinical setting, our proposed screening method remains exploratory and must be validated using standardized quantification methods for miRNAs such as qRT-PCR or digital PCR, before assessing their clinical potential. Moreover, miRNA read counts remain relatively low and confirmation by PCR (quantitative real-time or digital) will also be necessary to establish their plasma concentration. This validation is necessary to confirm the miRNAs detected by sequencing using another technique that is cheaper, faster and, above all, more suitable in a clinical setting. As both cohorts were of European ancestry, we cannot generalize our findings to other populations, including those that are at higher risk of GH [69]. Finally, further investigations on the specific miRNA targets are warranted to provide a better knowledge on their role in the pathophysiological mechanisms of GH.

## Conclusion

Circulating miR-208b-3p and miR-26a-1-3p in combination with first-trimester MAP show potential as early predictors of GH. Our approach, which integrates clinical risk factors and molecular biomarkers—akin to the Framingham algorithm for cardiovascular disease—represents a promising strategy for identifying women at increased risk of GH. However, the clinical utility of these miRNAs remains to be confirmed [70]. Additional studies are thus warranted to help better explain the pathophysiological processes related to GH that these miRNAs regulate.

## Electronic supplementary material

Below is the link to the electronic supplementary material.

**Supplementary Material 1**: **Supp Fig. 1**: Flowchart of the selected participants in the Gen3G cohort.

**Supplementary Material 2**: **Table 1**. Complete list of the 28 miRNAs associated with gestational hypertension onset in Gen3G.

## Data Availability

The datasets generated and analysed during the current study are available in the GEO repository, accession number Super Series GSE216998 https://www.ncbi.nlm.nih.gov/geo/query/acc.cgi?acc=GSE216998.

## Abbreviations

3D: Design, develop, discovery cohort
AIC: Akaike information criteria
AUC: Area under the curve
BMI: Body mass index
BP: Blood pressure
DBP: Diastolic blood pressure
C14MC: Chromosome 14 miRNA cluster
C19MC: Chromosome 19 miRNA cluster
CACNA1C: Calcium voltage-gated channel subunit alpha1 C
CACNB2: Calcium voltage-gated channel auxiliary subunit beta2
CDNK1A: Cyclin dependent kinase inhibitor 1 A
exceRpt: Extracellular RNA processing toolkit
FC: Fold change
FDR: False discovery rate
GDM: Gestational diabetes mellitus
Gen3G: Genetics of glucose regulation in gestation and growth cohort
GH: Gestational hypertension
HDP: Hypertensive disorders of pregnancy
IADPGS: International association of the diabetes and pregnancy study groups
LITAF: Lipopolysaccharide (LPS)-induced tumor necrosis factor α factor
LV: Left ventricular
MAP: Mean arterial pressure
miRNA: MicroRNA
mRNA: Messenger RNA
OGTT: Oral glucose tolerance test
PE: Preeclampsia
ROC: Receiver operating characteristic curve
SBP: Systolic blood pressure
SOGC: Society of obstetricians and gynaecologists of canada
TP53: Tumor protein p53
