# Supplementary material for: First trimester circulating miR-208b-3p and miR-26a-1-3p are relevant to the prediction of gestational hypertension
Source: BMC Pregnancy Childbirth. 2025 Mar 8;25:255. doi: 10.1186/s12884-025-07349-x (PMC11889763; doi:10.1186/s12884-025-07349-x)
Supplement: Supplementary file 1 — Supplementary Material 1: Supp Fig. 1: Flowchart of the selected participants in the Gen3G cohort. [file 12884_2025_7349_MOESM1_ESM.pdf]

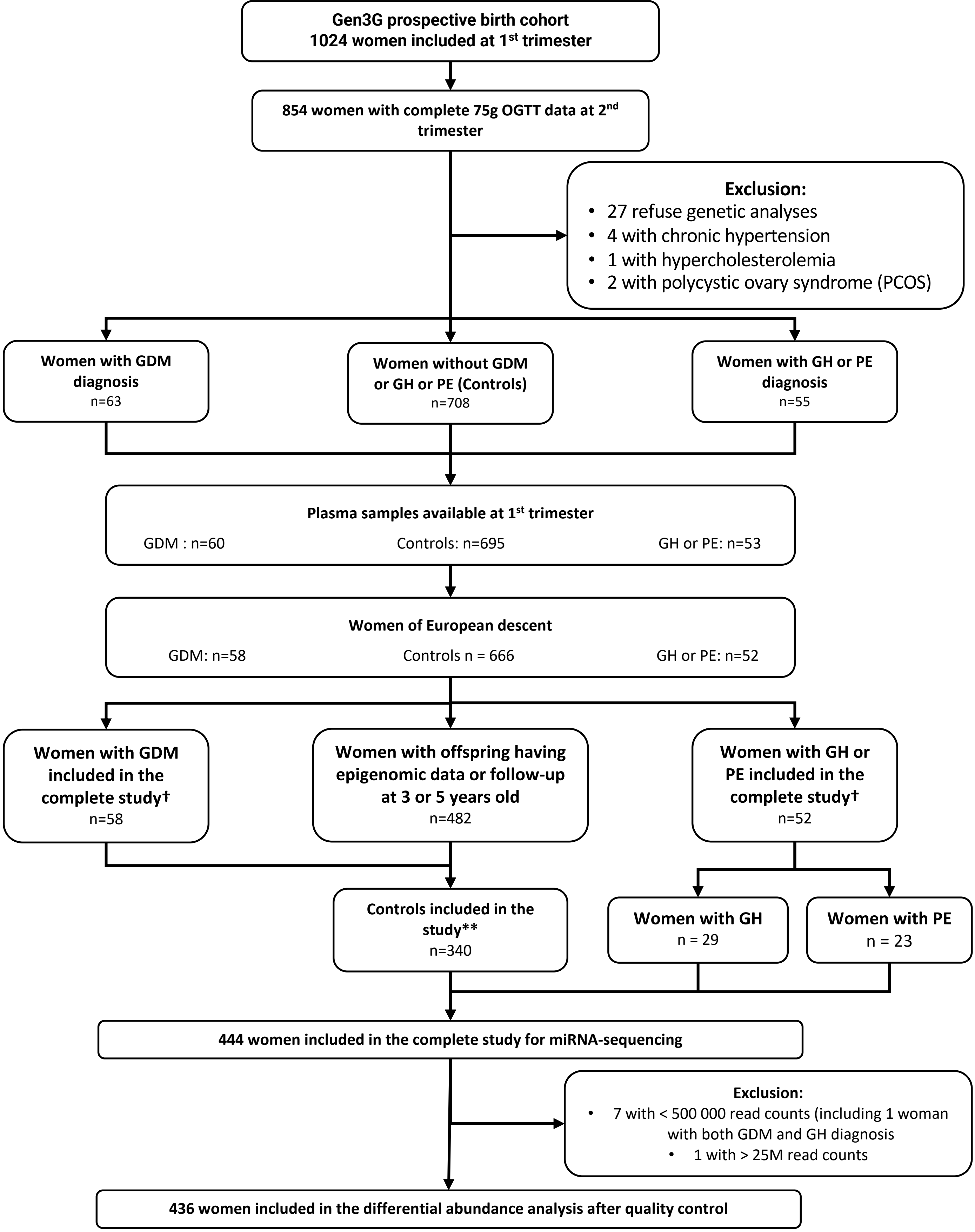

Supplementary Figure 1. Flowchart of the selected participants in the Gen3G cohort. This figure represents the flowchart for the selection of the participants in the Gen3G cohort. † 6 women having both GDM and GH or PE. ** Controls were selected by prioritizing women with offspring having both epigenomic data and subsequent follow-ups
